# Axial length changes in progressive and non-progressive myopic children in China

**DOI:** 10.1007/s00417-022-05901-5

**Published:** 2022-11-30

**Authors:** Jun Chen, Shang Liu, Zhuoting Zhu, Gabriella Bulloch, Thomas Naduvilath, Jingjing Wang, Linlin Du, Jinliuxing Yang, Bo Zhang, Haidong Zou, Xun Xu, Xiangui He

**Affiliations:** 1 Shanghai Eye Disease Prevention and Treatment Center, Shanghai Eye Hospital, Shanghai Vision Health Center & Shanghai Children Myopia Institute, Shanghai, 200030 China; 2 Department of Ophthalmology, Shanghai General Hospital, Shanghai Jiao Tong University, National Clinical Research Center for Eye Diseases, Center of Eye Shanghai Key Laboratory of Ocular Fundus Diseases, Shanghai Engineering Center for Visual Science and Photomedicine, Shanghai, 200080 China; 3 Centre for Eye Research Australia, Ophthalmology, University of Melbourne, Melbourne, Australia; 4 Brien Holden Vision Institute, Sydney, Australia; 5 School of Optometry and Vision Science, University of New South Wales, Sydney, Australia

**Keywords:** Non-progressive myopia, Progressive myopia, Axial length change, Children

## Abstract

**Purpose:**

Due to pubertal development and crystalline lens compensation, axial length (AL) continues to increase among non-progressive myopic children (absolute annual spherical equivalent (SE) progression less than 0.25 diopter), but the amount is unknown. This study was to investigate the cutoff of AL change to accurately differentiate between progressive and non-progressive myopes.

**Methods:**

A total of 8,546 myopic and treatment-naive children aged 6–10 years were enrolled from two cohort studies. AL with optical biometer and cycloplegic SE with auto refraction were evaluated at baseline and annually. Annual AL change was calculated, and the percentiles of annual axial elongation among progressive and non-progressive myopes were estimated by quantile regression with restricted cubic spline. Area under receiver-operating characteristic (ROC) curve (AUROC), positive predictive value (PPV), and negative predictive value (NPV) were applied to evaluate the accuracy of predicting progressive and non-progressive myopes.

**Results:**

Among 8,546 myopic children, 603 (7.06%) were non-progressive myopes. Annual AL changes among non-progressive myopes remained stable with the median annual change being 0.25 mm, while the median for progressive myopes decreased with age from 0.58 to 0.42 mm. AUROC for distinguishing between non-progressive and progressive myopes was 0.88 and was > 0.85 for each age group. Annual AL change, the cutoff of 0.20 mm/year, had significantly high PPV and NPV in predicting progressive myopes with high proportion of progressive myopes and non-progressive myopes with low proportions of progressive myopes.

**Conclusion:**

Myopic children with non-progressive status had markedly less axial elongation than progressive ones. AL changes with cutoff of 0.20 mm/year could differentiate between non-progressive and progressive status and may be an alternative for evaluating progressive status.


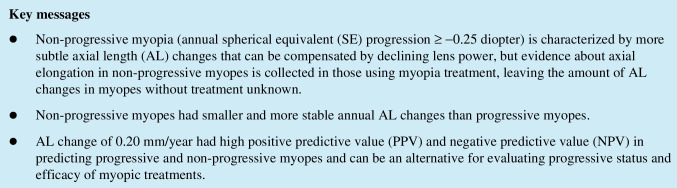


## Introduction

Myopia is generally characterized by abnormal axial elongation of the eye. Its prevalence in over 20% of the population has translated to a significant social and public health burden worldwide [1–3]. Axial myopia has the highest population burden among children and adolescents [4] and is diagnosed when axial length (AL) growth exceeds the loss of refractive power at the cornea and lens [5, 6]. Current studies suggest that visual impairment is more strongly associated with AL than refractive error [7–9], and excessive AL growth gives precipitate vision threatening complications including myopic retinopathy, retinal detachment, and glaucoma [10]. Therefore, AL growth is a good indicator of myopia progression that is detrimental to vision.

Myopia is considered irreversible; therefore, the main principle of myopia treatments is to inhibit axial growth [11, 12]. Myopia can be categorized as progressive or non-progressive, where the latter is characterized by more subtle AL changes that can be compensated by declining lens power. Thus, non-progressive myopes tend to have minimal effects on overall refractive error [13–15]. Differentiating non-progressive from progressive myopes is important for evaluating the effectiveness of myopia control treatments, which requires adjustment based on each individual’s responsiveness to treatment types. This is normally evaluated by changes in cycloplegic refraction where a 1-year absolute spherical equivalent (SE) change less than 0.25 D is considered non-progressive and SE change larger than 0.25 D is defined as progressive [13, 14].

Cycloplegic refraction albeit the gold standard is unsuitable for myopes who cannot undergo cycloplegia, like orthokeratology lens users [16]. Given the advantages of being non-invasive, having high accuracy and repeatability, AL evaluation is a useful alternative [12]. Tang et al. estimated that axial elongation in non-progressive myopes was 0.01 to 0.12 mm/year in boys and 0.003 to 0.11 mm/year in girls aged 6–18 years [14]. Non-progressive myopes aged 8 to 12 years showed an average axial elongation of 0.10 mm/year, according to the clinical trial MiSight [12], and this was 0.20 ± 0.25 mm when evaluated by Jiang et al. [17]. Currently, evidence about axial elongation in non-progressive myopes is collected in those using myopia treatment, leaving expected AL changes in myopes without treatment unknown. This is necessary to elucidate as this reference range can determine whether treatment efficacy is adequate. Moreover, such knowledge furthers understanding about the natural course of non-progressive myopes.

Therefore, this study recruited over 8,500 myopic children without myopia treatments aged 6–10 years from Shanghai, China, and aimed to investigate the axial elongation in progressive and non-progressive myopic children and identify the cutoff as well as evaluate its accuracy to differentiate between progressive and non-progressive myopes.

## Methods

### Participants

Data on cycloplegic SE, AL, corneal curvature radius (CCR), and demographic characteristics among 8,546 myopic children aged 6–10 years were collected from two independent prospective studies conducted in Shanghai. The first longitudinal study was conducted from 2013 to 2016 and the second, from 2016 to 2020. Each study collected patient questionnaires and ocular examinations at baseline and annual follow-up. Details of the design and methodology of each study are reported elsewhere [18, 19].

The inclusion criteria of individuals were as follows: (1) with complete information including cycloplegic SE, AL, corneal curvature radius, demographic characteristics, and myopia treatments; (2) at least one consecutive follow-up visit; (3) without taking myopia treatments at the initial recruitment and within the two consecutive visits. Individuals with outliers or unlogic values were excluded. Information was de-identified for analysis, and written informed consent was provided by children’s parents or other guardians in the two studies. This study adhered to the Declaration of Helsinki and was approved by Shanghai General Hospital Ethics Committee (No. 2016KY138).

### Measurements

Demographics including age, gender, and ethnicity of each participant were retrieved from questionnaires, and baseline ocular parameters including AL, CCR, and SE were acquired from examination recordings. Both longitudinal studies employed experienced optometrists and assistants with similar manual instructions for ocular examinations. AL was measured by optical biometer (IOL Master; version 5.02; Carl Zeiss, Jena, Germany), spherical refraction measurements were taken following cycloplegia using 0.5% proparacaine hydrochloride (Alcaine; Alcon, Fort Worth, TX, USA) followed by 2 drops of 1% cyclopentolate (Cyclogyl; Alcon) 5 min apart. Once the pupil size was 6 mm or greater and light reflex was absent, spherical refraction was measured using a desk-mounted auto-refractor (KR-8900; Topcon, Tokyo, Japan).

### Definitions

The refractive state was defined as myopia when SE ≤  − 0.50 D, and non-myopia was considered at SE >  − 0.50D. AL was defined as the distance from the surface of the anterior cornea to the retinal pigment epithelium (RPE). CCR was calculated as the mean of the flattest and steepest radii. SE progression, CCR change, and axial elongation were calculated by subtracting their previous values a year earlier.

According to the definition of non-progressive myopes in previous studies [12, 14] and the precision of cycloplegic refractive error (± 0.25 D), 1-year absolute SE progression less than 0.25 D was considered as non-progressive myopes. 1-year absolute SE progression larger than 0.25 D was defined as progressive myopes.

### Statistical analyses

Only data from the right eyes were assessed. The Kolmogorov–Smirnov test assessed the normal distribution of ocular parameters. Continuous variables were expressed as a mean and standard deviation for normal distribution, median with quantiles for skewed distribution, and frequencies with proportions for categorical data. Two-tailed *t*-tests or Wilcoxon tests investigated gender differences given the distribution of AL change. Trend analyses with general linear regression tested variations between ages. Quantile regression with restricted cubic splines, recommended by the World Health Organization for modeling growth velocity curves [20], was applied to test nonlinear associations between age and axial elongation and estimated annual AL changes. Area under receiver-operating characteristic (ROC) curve (AUROC) evaluated the classification of progressive and non-progressive myopes. Different annual AL changes were evaluated and calculated for cutoffs by taking into account the distribution and measurement error. Positive predictive value (PPV) and negative predictive value (NPV) of cutoffs were applied to evaluate the accuracy of predicting progressive and non-progressive myopes. Taking PPV and NPV into consideration together, the value with NPV and PPV both larger than 80% was selected as the appropriate cutoff. Statistical analyses were conducted with SAS version 9.4 (SAS Institute Inc.) and R 4.1.0 (R Core Team, 2022).

## Results

### General characteristics

A total of 8,546 myopic children were included for analyses. Mean age was 8.21 ± 1.09 years, and 52.8% of participants were male. Among all myopes, the average baseline SE was − 1.88 ± 1.30 D with an average annual change of − 0.95 ± 0.48 D. Mean baseline AL was 24.17 ± 0.90 mm, and annual AL change was 0.47 ± 0.21 mm. Baseline CCR was 7.82 ± 0.26 mm and remained stable during the follow-up. The annual body height change was 6.14 ± 2.72 cm. Among the included participants, 603 (7.06%) were non-progressive. Table 1 summarizes the baseline characteristics of progressive and non-progressive myopic children. Non-progressive myopes were older in age and higher in base body height and body height change than progressive myopes (*P* < 0.05). Progressive myopes had smaller baseline SE, more SE progression, and AL change than non-progressive myopes (*P* < 0.001).

**Table 1:** Baseline characteristics of myopic eyes among progressive and non-progressive myopes

Characteristics	Total (*n* = 8546)	Progressive myopes (*n* = 7943)	Non-progressive myopes (*n* = 603)
Age (yrs), mean (SD)	8.21 (1.09)	8.19 (1.10)	8.46 (1.00)*
Boys, *N* (%)	4512 (52.80)	4145 (52.18)	367 (60.86)**
SE (D)			
Baseline, mean (SD)	− 1.88 (1.30)	− 1.91 (1.30)	− 1.61 (1.26)**
1-year change, mean (SD)	− 0.95 (0.48)	− 1.02 (0.42)	− 0.14 (0.14)**
AL (mm)			
Baseline, mean (SD)	24.17 (0.90)	24.17 (0.90)	24.23 (0.83)
1-year change, mean (SD)	0.47 (0.21)	0.49 (0.21)	0.25 (0.11)**
Baseline CCR (mm), mean (SD)	7.82 (0.26)	7.82 (0.26)	7.83 (0.27)
Body height (cm)			
Baseline, mean (SD)	139.81 (8.85)	139.74 (8.89)	140.71 (8.32)*
1-year change, mean (SD)	6.14 (2.72)	6.16 (2.74)	5.93 (2.45)*

*IQR*, interquartile range; *SE*, spherical equivalent; *AL*, axial length; *CCR*, corneal curvature radius

^*^P < 0.05, **P < 0.001 for the comparison between progressive and non-progressive myopes

### Annual AL changes in progressive and non-progressive myopes

**Fig. 1:**
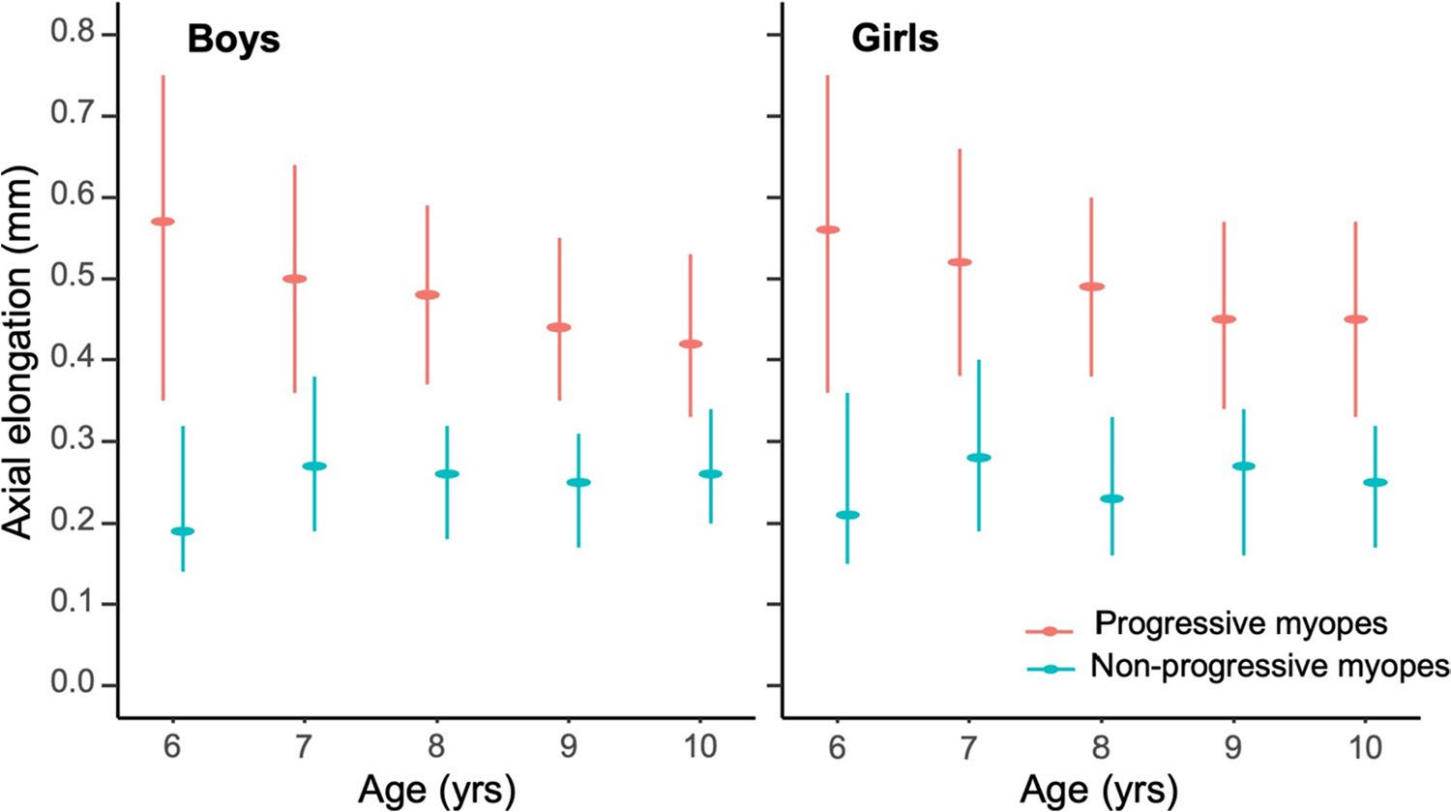
Age-specific annual axial length change among myopic boys and girls. Dot indicated the median and the error bar for the 25th and 75th percentiles, respectively

Annual AL changes stratified for age is shown in Table 2 and Fig. 1. As age increased from 6 to 10 years old, annual AL changes among progressive myopes gradually decreased from 0.57 (IQR: 0.35 ~ 0.75) to 0.42 mm/year (IQR: 0.33 ~ 0.53) in boys, from 0.56 (IQR: 0.36 ~ 0.75) to 0.45 mm/year (IQR: 0.33 ~ 0.57) in girls (*P* < 0.001), and average change was 0.49 mm/year. The AL changes among non-progressive myopes did not vary with age, and the median change was 0.25 mm/year (*P* = 0.999 and *P* = 0.787, respectively).

**Table 2:** Age-specific axial elongation among boys and girls aged 6 to 10

Age (yrs)	Boys				Girls			
	Progressive myopes^ab^		Non-progressive myopes^cd^		Progressive myopes^ab^		Non-progressive myopes^cd^	
	*n*	Median (IQR)	*n*	Median (IQR)	*n*	Median (IQR)	*n*	Median (IQR)
6	303	0.57 (0.35 ~ 0.75)	8	0.19 (0.14 ~ 0.32)	264	0.56 (0.36 ~ 0.75)	8	0.21 (0.15 ~ 0.36)
7	871	0.50 (0.36 ~ 0.64)	57	0.27 (0.19 ~ 0.38)	755	0.52 (0.38 ~ 0.66)	30	0.28 (0.19 ~ 0.40)
8	1185	0.48 (0.37 ~ 0.59)	118	0.26 (0.18 ~ 0.32)	1086	0.49 (0.38 ~ 0.60)	75	0.23 (0.16 ~ 0.33)
9	1360	0.44 (0.35 ~ 0.55)	124	0.25 (0.17 ~ 0.31)	1292	0.45 (0.34 ~ 0.57)	94	0.27 (0.16 ~ 0.34)
10	426	0.42 (0.33 ~ 0.53)	60	0.26 (0.20 ~ 0.34)	401	0.45 (0.33 ~ 0.57)	29	0.25 (0.17 ~ 0.32)
Total	4145	0.47 (0.35 ~ 0.60)	367	0.25 (0.18 ~ 0.32)	3798	0.48 (0.36 ~ 0.61)	236	0.25 (0.16 ~ 0.34)

*IQR*, interquartile range

^a^Wilcoxon tests for progressive myopes between boys and girls were statistically different at the age of 7, 8, and 9 (*P* = 0.006, *P* = 0.041, and *P* = 0.021, respectively), but not statistically different at the age of 6 and 10 (*P* = 0.585 and *P* = 0.103, respectively)

^b^Trend analyses for age-specific progressive myopes within the strata of boys and girls were statistically different (*P* < 0.001 and *P* < 0.001, respectively)

^c^Wilcoxon tests for age-specific non-progressive myopes between boys and girls were not statistically different (from 6 to 10, *P* = 0.880, *P* = 0.113, *P* = 0.764, *P* = 0.609, and *P* = 0.523, respectively)

^d^Trend analyses for age-specific non-progressive myopes within the strata of boys and girls were not statistically different (*P* = 0.999 and *P* = 0.787, respectively)

Annual AL changes among non-progressive myopes for each age according to gender were not statistically different (*P* = 0.880, 0.113, 0.764, 0.609, and 0.523, between gender for each age group, respectively). Annual AL changes among progressive myopes between genders were statistically different at the age of 7, 8, and 9 (*P* = 0.006, *P* = 0.041, and *P* = 0.021, respectively), but not statistically different at ages 6 and 10 (*P* = 0.585 and *P* = 0.103, respectively).

### Estimated annual AL changes

**Fig. 2:**
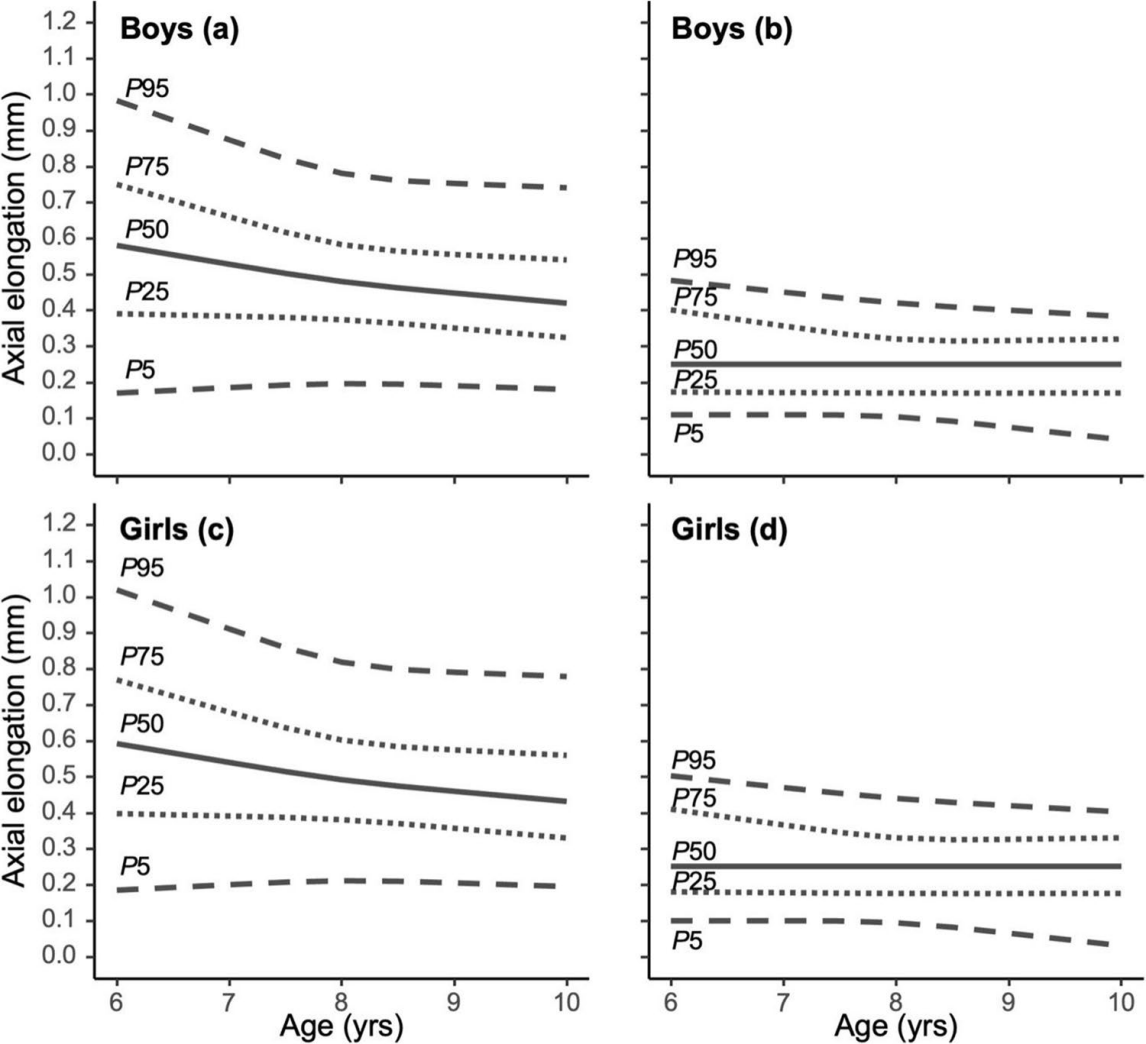
Percentiles of age-specific annual axial length change in myopic boys and girls. **a** Progressive myopes in boys; **b** non-progressive myopes in boys; **c** progressive myopes in girls; **d** non-progressive myopes in girls

Table 3 and Fig. 2 estimate the percentiles of annual axial changes among progressive and non-progressive myopes for boys and girls with age. For progressive myopes, percentiles above the 50th for AL growth were associated with decreasing age in boys and girls, while 25th and below percentiles had more stable AL change across gender and age. For non-progressive myopes, AL percentiles of 75th and above were negatively associated with age, but other percentiles remained stable across gender and age. The percentiles of annual AL changes among non-progressive myopes were similar between boys and girls, and the 50th percentile among both genders fluctuated around 0.25 mm.

**Table 3:** Age-specific estimated percentiles of axial elongation among myopic boys and girls aged 6 to 10

Age (yrs)	Boys										Girls									
	Progressive myopes					Non-progressive myopes					Progressive myopes					Non-progressive myopes				
	*P5*	*P25*	*P50*	*P75*	*P95*	*P5*	*P25*	*P50*	*P75*	*P95*	*P5*	*P25*	*P50*	*P75*	*P95*	*P5*	*P25*	*P50*	*P75*	*P95*
6	0.17	0.39	0.58	0.75	0.98	0.11	0.17	0.25	0.40	0.48	0.19	0.40	0.59	0.77	1.02	0.10	0.18	0.25	0.41	0.50
7	0.19	0.38	0.53	0.66	0.87	0.11	0.17	0.25	0.36	0.45	0.20	0.39	0.54	0.68	0.91	0.10	0.18	0.25	0.37	0.47
8	0.20	0.37	0.48	0.58	0.78	0.10	0.17	0.25	0.32	0.42	0.21	0.38	0.49	0.60	0.82	0.09	0.18	0.25	0.33	0.44
9	0.19	0.35	0.45	0.55	0.75	0.07	0.17	0.25	0.32	0.40	0.21	0.36	0.46	0.57	0.79	0.06	0.18	0.25	0.33	0.42
10	0.18	0.32	0.42	0.54	0.74	0.04	0.17	0.25	0.32	0.38	0.20	0.33	0.43	0.56	0.78	0.03	0.18	0.25	0.33	0.40

Quantile regression with restricted cubic splines was applied for estimating the age-specific percentiles

### Accuracy for differentiating between progressive and non-progressive myopes

**Fig. 3:**
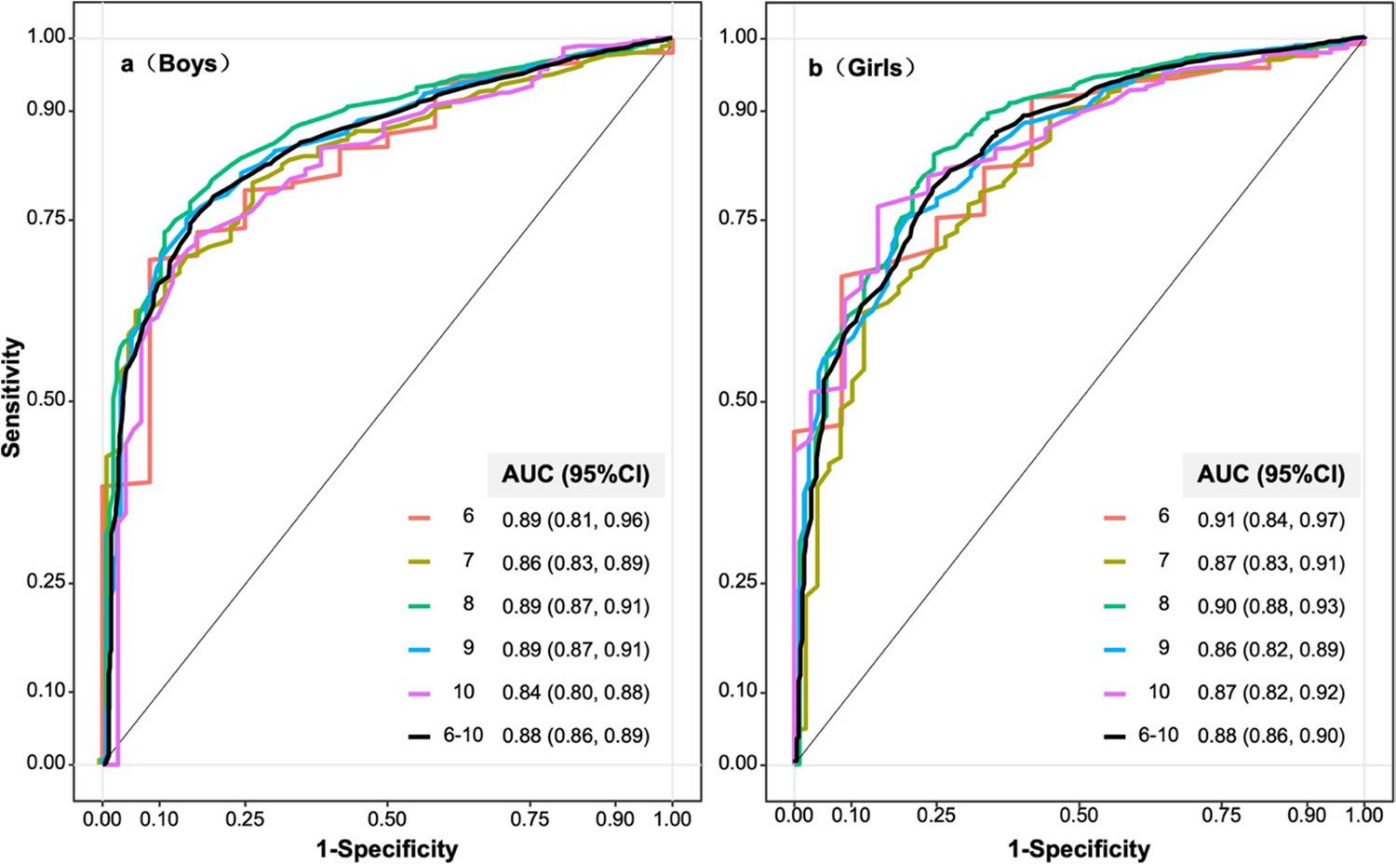
Receiver-operating characteristics curves for predicting progressive and non-progressive myopes among girls and boys by age. AUC, area under the curve; 95% CI, 95% confidence interval

**Fig. 4:**
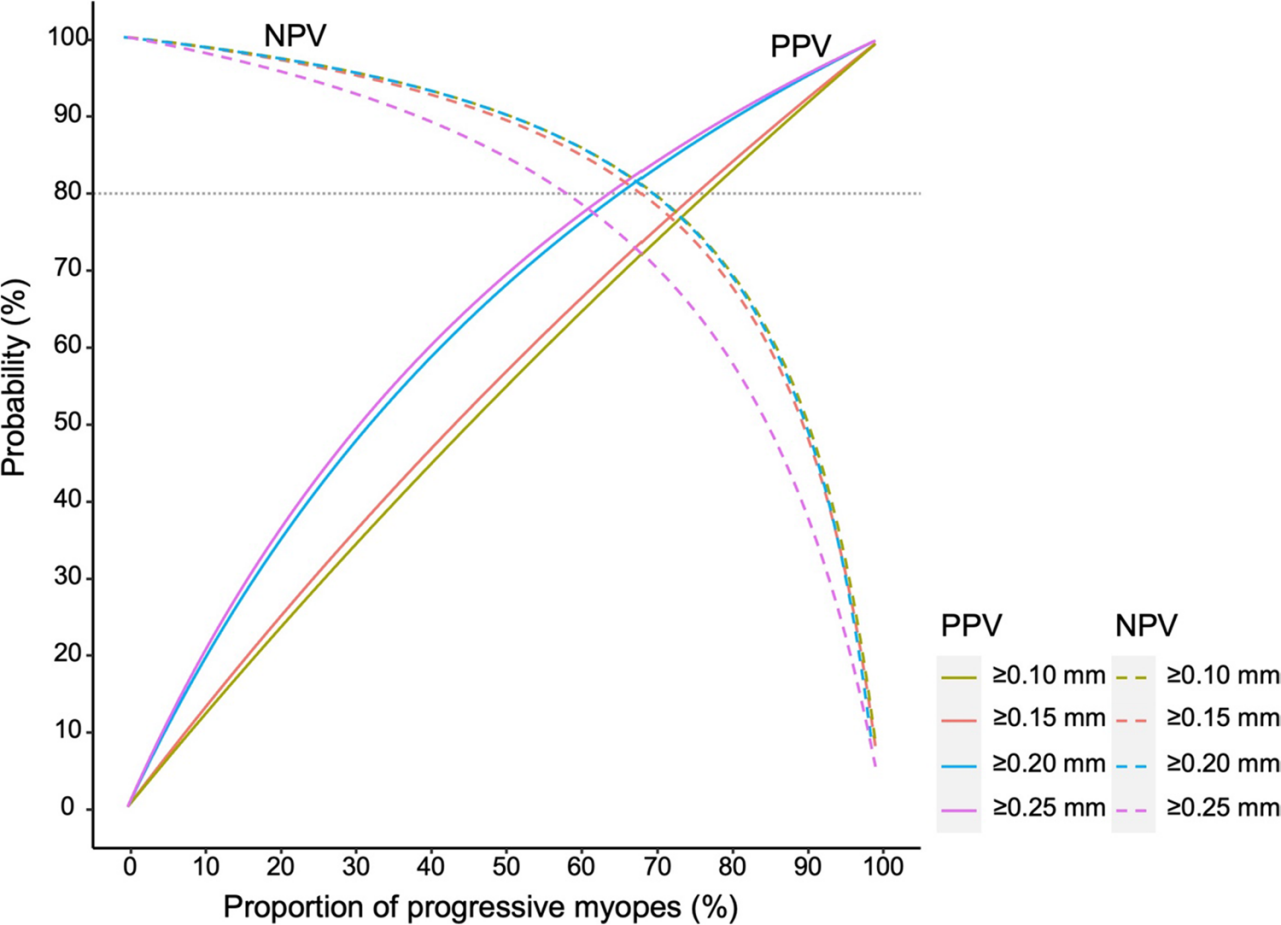
Positive predictive value and negative predictive value for various cutoffs of axial length change. PPV, positive predictive value; NPV, negative predictive value; proportion, the percentage of progressive myopes among all myopes; the horizontal gray line for the prediction accuracy of 80%

The AUROC for annual AL changes to distinguish between non-progressive and progressive myopes was 0.88 (Fig. 3). Age-specific AUROCs for distinguishing non-progressive and progressive myopes all exceeded 0.85. Given the percentiles of annual AL changes and AUROC were similar across genders and ages, Table 4 and Fig. 4 presented the whole prediction accuracy of annual AL changes, and the results indicated that the prediction accuracy mainly depended on the proportion of progressive myopes among the whole myopes. PPV increased with the proportions, while NPV decreased with the proportions. For example, at the cutoff of ≥ 0.10 mm/year, when the proportion of progressive myopes increased from 10 to 90%, the PPV increased from 13 to 93%, and the NPV decreased from 99 to 45%. Taking the PPV and NPV into consideration together, at a lower proportion of progressive myopes, NPV was preferred and the cutoff of 0.20 mm/year performed as good as 0.10 mm/year and 0.15 mm/year. While at the higher proportion, PPV was preferred, and the cutoff of 0.20 mm/year was comparable with 0.25 mm/year.

**Table 4:** The accuracy of various cutoffs of annual axial length change for differentiating progressive and non-progressive myopes

Cutoff (mm/year)	Proportion (%)	Sensitivity (%)	Specificity (%)	PPV (%)	NPV (%)
≥ 0.10					
	10	96	30	13	99
	50	96	30	58	88
	90	96	30	93	45
≥ 0.15					
	10	95	43	16	99
	50	95	43	63	90
	90	95	43	94	49
≥ 0.20					
	10	93	55	19	99
	50	93	55	67	89
	90	93	55	95	47
≥ 0.25					
	10	89	60	20	98
	50	89	60	69	85
	90	89	60	95	38

*PPV*, positive predictive value; *NPV*, negative predictive value; *Proportion*, the percentage of progressive myopes among all myopes; ≥ 0.10, ≥ 0.15, ≥ 0.20, and ≥ 0.25 indicated different cutoffs of annual axial length change (mm/year)

## Discussion

This study combined two large prospective studies and enrolled over 8,500 myopes. In this cohort, 603 participants (7.06%) were deemed non-progressive myopes and exhibited stable annual rates of axial elongation while the progressive myopes demonstrated significantly higher annual axial elongation. We found that AL changes of 0.20 mm/year had good PPV and NPV for predicting progressive and non-progressive myopes given the proportions of progressive myopes among the whole myopes. This suggests that annual AL changes can accurately predict progressive status and act as a proxy for evaluating the clinical efficacy of myopic treatments.

Our findings suggest that axial elongation in non-progressive myopes is not influenced by gender, while AL elongation was significantly larger in girls with progressive myopia. The results are consistent with previous trends found in emmetropes, which found that gender differences were not associated with axial length changes in normal eye development [21, 22]. Likewise, the influence of the female gender on progressive axial elongation is consistent with the large multi-center Collaborative Longitudinal Evaluation of Ethnicity and Refractive Error (CLEERE), where annual axial elongation was significantly faster for girls [6, 23]. Li et al., who also noted this gender effect in low and high myopes, observed girls generally had smaller eyes with steeper corneal curvatures [6]. Although they hypothesized this might inherently predispose females to longer anterior segments to compensate for long axial lengths, our larger cohort did not observe these gender differences in non-progressive myopes. As the participants were pre-teen, it is unlikely hormones played significant roles in the gender discrepancies observed in progressive myopes. More likely, the superimposition of environmental factors on genetic/physiological determinants is a tempting explanation for this association with gender, as girls spend less time outdoors than boys when playing, and are generally more studious in their younger years [24, 25]. As light exposure is a known modifiable risk factor for myopia, it is possible that the norms of gender play influence behaviors that may ultimately predispose girls to more progressive myopia.

Furthermore, we observed the percentiles of non-progressive myopes between the 25th and 50th range had axial elongations that remained stable with age, although their magnitudes were still too large to be considered normal physiological axial elongation in emmetropic children. The physiological elongation among emmetropes aged 8–13 years in the Singapore Cohort Study of the Risk Factors for Myopia (SCORM) study fluctuated around 0.14 mm/year [15] and was 0.16 mm/year in the CLEERE study among emmetropes aged 6–9 years [23]. Among two studies evaluating AL in non-progressive myopes with myopic treatments [12, 14], the annual axial elongation varied from 0.01 to 0.12 mm/year, while this average was 0.25 mm/year in the current study evaluating similar myopes. This finding indicates that non-progressive myopes are not predisposed to AL changes consistent with emmetropization and that myopic treatment is required in these individuals to correct annual AL change to a physiologic range. The annual AL change percentiles provide an expected range of axial elongation among non-progressive myopes, and these ranges may be referred to in future studies wishing to evaluate the efficacy of treatments. For example, an annual AL > 0.25 mm/year approaches AL changes which describe no curtailing from the natural history of non-progressive myopia and suggests an inadequate myopia treatment response.

Our study observed that high AUROCs which determined annual AL change also had a good ability to differentiate between progressive and non-progressive myopes. Furthermore, promising PPV and NPV indicated that annual AL changes had high accuracy to predict progressive and non-progressive myopes. Currently, few studies have theorized cutoffs that can distinguish non-progressive and progressive myopes. In a 3-year randomized clinical trial of MiSight lenses, for participants taking MiSight lenses with SE progression less than 0.25 D, the annual axial elongation was 0.10 mm, and most participants were non-progressive myopes [13]. Given the proportions of progressive myopes varied across different scenarios, PPV and NPV should be taken together into consideration, and the cutoff of 0.20 mm/year was appropriate to distinguish between progressive and non-progressive myopes. For example, regarding the efficacy of current myopia treatments is generally good [16], most were non-progressive myopes, and the cutoff of 0.20 mm/year is good for identifying non-progressive myopes. However, among myopes without myopia treatment, most were progressive myopes, and the cutoff of 0.20 mm/year had high accuracy for identifying progressive myopes. It is our opinion that AL changes can be an useful proxy for evaluating the progressive status in practice, especially for those unable to undergo cycloplegia for full refractive examinations, like orthokeratology users.

Although this study provides important reference values for axial elongation in non-progressive myopes, several limitations should be acknowledged. First, although our sample sizes are larger than the populations of the SCORM, MiSight, and CLEER studies, they are still too small to generalize for all myopes. A population-based myopia study is yet to characterize these findings in non-progressive myopes. Second, the age range did not include children older than 10, so the current findings cannot be confidently generalized to children aged 11 years or older. Third, given all children were enrolled from Shanghai, there may be differences in children from other geographic and ethnic populations. Last but not the least, children with incomplete follow-up information were excluded from analysis, and this may induce potential selection bias in our study. Future studies could include a multicentered approach, with myopes encompassing a larger age range and longer follow-up times, which would provide more generalizable results.

In conclusion, non-progressive myopes still had significant axial elongation but these were distinguished from their progressive myopes and emmetropes in the literature. Annual axial elongation had high accuracy in distinguishing between progressive and non-progressive myopes. This suggests that annual AL changes may be useful for evaluating myopia progression and the efficacy of myopia treatments in clinical practice.
